# High-Frequency Functional Trajectories Predict Depressive Worsening in Singapore’s Community-Dwelling Older Adults

**DOI:** 10.3390/healthcare14050629

**Published:** 2026-03-02

**Authors:** Kaung H. T. Salai, Yi Wen Tan, Grace Cheong, Paulin Straughan

**Affiliations:** Centre for Research on Successful Ageing, Singapore Management University, Singapore 179873, Singapore; ywtan@smu.edu.sg (Y.W.T.); llcheong@smu.edu.sg (G.C.); paulints@smu.edu.sg (P.S.)

**Keywords:** functional difficulty, depression, k-means longitudinal clustering, ageing population, superaged societies

## Abstract

**Highlights:**

**What is the main finding?**

- Using a data-driven longitudinal clustering approach, this study found that older Singaporeans with greater functional difficulty trajectories faced a significantly higher risk of worsening depression within a one to two-year period.

**What is the implications of the main finding?**

- The findings emphasise the importance of early rehabilitation and supportive services for older adults experiencing rapid functional difficulty as an effective approach to lower late-life depression rates.

**Abstract:**

**Background/Objectives**: Functional difficulty and depression often coexist in older adults, yet local Singapore-based research often lacks detailed temporal resolution due to heterogeneity in ageing. This study employs non-parametric, data-driven longitudinal clustering to analyse functional trajectories and their association with depression, using high-frequency data to pinpoint key intervention periods. **Methods**: Data were drawn from 4273 community-dwelling older adults from Singapore Life Panel^®^ (2020–2024). Participants completed quarterly self-reported assessments of ADL, IADL and depressive symptoms (8-item CES-D). We employed k-means longitudinal clustering (kml) to identify functional trajectory groups and Cox regression to evaluate the hazard of increased depression (≥5-point increase in CES-D). **Results**: Three distinct trajectories emerged for both ADL and IADL (Stable, Medium increase in difficulty, High increase in difficulty). In fully adjusted Cox models, Medium and High clusters had higher hazard ratios for increased depression than Stable (ADL: HR 1.71 and 2.37; IADL: HR 1.60 and 2.20). Median time-to-event was not reached in the Stable group and occurred earlier in Medium/High clusters (ADL: 3.25 years and 1.75 years; IADL: 4.0 years and 2.1 years). The High cluster, comprising older and socioeconomically disadvantaged individuals, exhibited worse baseline health and psychosocial factor scores. Depression scores escalated in the Medium and High groups. **Conclusions**: Rapid functional difficulty acts as a precursor to worsening depressive symptoms. Routine monitoring of functional trajectories offers a strategic window for proactive mental health interventions in at-risk older adults.

## 1. Introduction

Population ageing and extended lifespans have brought increasing concern about functional difficulty and mental well-being in later life. In Singapore, 20.7% of its citizens were aged 65 and older as of June 2025, and the proportion is expected to surpass the super-aged threshold of 21.0% by 2030 [1]. This demographic shift is accompanied by a rise in chronic health conditions, a loss of functional independence, and an increase in mental health challenges. One particularly salient concern is late-life depression. The recent Well-being of Singapore Elderly (WiSE) national survey reported 4.4% of older Singaporeans meeting the criteria for depression syndrome, while 11.9% experiencing subsyndromal depression [2]. Depression in older adults is associated with disability, poorer quality of life, and higher healthcare costs [2,3]. Although this prevalence appears low, it increases the burden of care for late-life depression in an ageing society. This burden is exacerbated by shrinking family sizes; the Old-Age support Ratio in Singapore will drop from 7.7 in 2010 to 2.2 in 2030, signalling a looming scarcity of home-based caregivers [4,5]. It is therefore critical to understand how physical functional changes intertwine with mental health, ensuring that increased longevity is accompanied by an extended health span.

Numerous longitudinal studies reveal a bidirectional relationship between functional difficulty and depressive symptoms in later life. New or worsening difficulties in activities of daily living (ADLs) and instrumental ADLs (IADLs) predict subsequent increases in depressive symptoms, while pre-existing depressive symptoms modestly elevate the risk of future functional difficulties [6,7]. Although functional difficulties generally predict depression, existing research in Singapore often lacks the longitudinal resolution to pinpoint exactly when depressive symptoms are likely to worsen in individuals experiencing functional difficulties. Local studies often rely on limited follow-up time-points, such as annual or biennial panels, which can obscure the temporal dynamics of the ADLs/IADLs–depression link [8,9,10,11]. Events over months are often aggregated, leading to interval censoring and masking of finer dynamics. The assumption of smooth trajectories over extended intervals can obscure heterogeneous effects and limit the ability to detect important within-year fluctuations. Without granular data on the rate and onset of these changes, it becomes difficult to determine if depression manifests gradually or follows a more acute onset in response to specific types of functional loss. Addressing this requires a shift from simple prevalence statistics toward higher-frequency assessments. Such approaches provide greater precision, allowing for a more accurate representation of dynamic pathways and capturing the variability of change within shorter timeframes to clarify the precise temporal sequence of difficulty.

Uncovering these temporal patterns requires a methodological approach that respects the heterogeneity of the ageing process. Functional ageing is not uniform; rather, it encompasses a diverse range of trajectories [12,13]. For instance, longitudinal studies in the US and Japan consistently distinguished between a majority maintaining independence and smaller subgroups experiencing progressive or persistent impairment [14,15]. These findings highlight that while many participants remain in the robust category, a minority transition to greater dependency. This difficulty is rarely a smooth, monotonic process. Instead, it is frequently punctuated by acute health events, such as falls, hospitalisations, or sudden onset of illness, that result in jagged, irregular difficulties rather than a gradual slope [16]. Capturing these irregularities is essential, but standard trajectory-mixture approaches such as Latent Class Growth Analysis (LCGA) and Group-Based Trajectory Models (GBTM) typically represent each latent class using prespecified parametric functions of time (often linear, quadratic, or spline-based) [13,17]. Consequently, the resulting class trajectories can be artificially smooth, potentially obscuring within-class heterogeneity [18]. This constraint reduces sensitivity to short-term fluctuations or abrupt changes, often causing acute patterns to be absorbed into broader gradual-change categories [19,20].

To better capture irregular functional change, k-means for longitudinal data (kml) is a nonparametric alternative that applies a hill-climbing algorithm adapted from k-means clustering. This approach analyses full longitudinal trajectories using Euclidean distance on a standardised time grid, allowing it to avoid the limitations of predetermined parametric shapes within-class [21] and capture irregular departures from simple parametric patterns. The kml algorithm enhances robustness by utilising multiple random starts and retaining the solution that optimises an internal clustering criterion, such as the Calinski-Harabasz index [21]. The utility of this method is increasingly recognised in epidemiological research, with successful applications that map distinct clinical phenotypes and symptom trajectories across diverse health domains [22,23].

To address these gaps, the current study employs data-driven kml clustering to analyse quarterly ADL and IADL scores, identifying groups of functional trajectories among older Singaporeans. Because functional changes and depression are embedded in psychosocial and cognitive contexts, we accounted for baseline social isolation, social support and engagement and cognitive status—factors associated with both functional disability and depressive outcomes [17,24,25,26,27]. Drawing on disablement and activity-restriction frameworks, we posit that functional deterioration precedes depressive worsening by eroding autonomy/mastery and restricting engagement in valued activities and social roles [28,29]. We therefore hypothesised that both ADL and IADL trajectories would separate into multiple distinct clusters, and that clusters characterised by increased functional difficulty would experience a significantly increased risk of worsening depressive symptoms. By integrating this flexible trajectory modelling with prospective depression outcomes, we aim to pinpoint time windows when mental health prevention and support may be most impactful.

## 2. Materials and Methods

### 2.1. Data

This research utilised data from the Singapore Life Panel^®^ (SLP), a high-frequency longitudinal survey targeting older residents aged 50 to 70 years and their spouses [30]. Participants could complete surveys online, via telephone, or through in-person interviews. To accommodate Singapore’s diverse ethnic composition, surveys were administered in English, Chinese, Malay, and Tamil, with respondents receiving vouchers as compensation. Recruitment commenced in 2015, initially reaching approximately 11,500 households, and the panel has consistently achieved an average response rate of 70.0% since inception. In 2020, the SLP underwent an infrastructural update, followed by a period of renewal from 2021 to 2023. After accounting for attrition, mortality, and data quality filtering, a cohort of 4273 respondents consistently provided quarterly responses on activities of daily living (ADL), instrumental activities of daily living (IADL), and depression items from November 2020 through November 2024 (see Supplementary Figure S1). Because longitudinal clustering requires comparable trajectories over time, kml clustering was conducted using complete quarterly ADL and IADL trajectories within the analytic cohort; no imputation of ADL/IADL values was performed for clustering. Additionally, these participants completed baseline assessments of social support, social engagement, social isolation and cognitive items.

### 2.2. Measurements

#### 2.2.1. Activities of Daily Living (ADL) and Instrumental Activities of Daily Living (IADL)

Participants’ difficulties with ADLs and IADLs were assessed through self-report measures that were validated for the Singapore population (see Supplementary Table S1) [31,32]. For ADLs, participants rated the difficulty of completing six activities on a scale from 1 (Not at all difficult) to 4 (Unable to perform). The overall ADL score for each individual was calculated by summing responses across all six ADL items, with higher scores indicating greater difficulty. Similarly, IADLs were evaluated by participants’ self-rated difficulty across eight instrumental activities using the same scale. The total IADL score was obtained by summing the eight items, with higher scores indicating greater difficulty performing these tasks.

#### 2.2.2. Depression

In this research, participants’ levels of depression were assessed through their self-reporting on the eight-item version of the Centre for Epidemiologic Studies-Depression Scale (CES-D), as included in the European Social Survey (ESS) and referenced by Van de Velde et al. (2009) [33]. Participants reflected on the frequency of experiencing various depressive symptoms over the past week (i.e., felt sad, happiness, loneliness, depressed, everything you did was an effort, sleep was restless, enjoyment of life, and could not get going). Item responses were provided on a six-point scale, ranging from 1 (none of the time) to 6 (all of the time). To ensure consistent scoring, the positively worded items were reverse-coded so that higher total scores uniformly indicated more severe depressive symptoms.

For each participant, we defined “increased depression” as the first follow-up wave with a ≥5-point increase from baseline. This threshold corresponded to the 95th percentile of the observed change in our cohort [34], capturing marked within-cohort worsening rather than a clinically validated cutoff. Because this study used a self-reported 8-item CES-D variant and did not include CES-D-20 administration or clinical depression diagnoses, we were unable to calibrate change scores to established clinical cut-points or symptom-severity categories. In addition, our CES-D-8 scoring differs from those of commonly used short-form versions in the literature, limiting direct comparability and preventing the defensible use of published thresholds. We therefore operationalised “increased depression” using a distribution-based definition (95th percentile of observed change from baseline) to capture marked symptom worsening within this cohort. We reported sensitivity analyses using a stricter threshold (≥10-point increase) and Firth penalised Cox regression models (see Supplementary Tables S2–S4).

#### 2.2.3. Covariates

Covariates included in this study were age, gender, education, marital status, housing, the number of chronic diseases, social support, isolation and engagement, and cognitive failures scores. Demographic covariates (namely age, gender, education, marital status, and housing) were collected at baseline (November 2020). Education was measured on a scale from 1 (No formal schooling/primary education) to 4 (University education). Housing was measured on a scale from 1 (1–3-room HDB flat) to 3 (Private Housing), as housing type is commonly used to approximate socioeconomic status because it correlates with income [35].

Perceived Social Support was measured in November 2020, using seven items from the Medical Outcomes Study Social Support Survey [36] (see Supplementary Table S1). These items assessed how often participants felt they were supported emotionally (e.g., “someone to confide in or talk to about your problems”) and in their daily provisions (e.g., “someone to prepare meals if you are unable to do so”) on a five-point scale (1 = none of the time, 5 = all the time). A final social support index was derived by adding the scores of all seven items. Perceived social isolation was measured in November 2020 by asking the respondents, “How often do you feel isolated from others?” using a five-point scale (1 = none of the time, 5 = all the time).

Participants’ social engagement was assessed in November 2020 by recording how often they participated in seven different activities over the previous month (see Supplementary Table S1). The selection of items was based on the taxonomy of social activities developed by Levasseur, Richard, Gauvin, and Raymond [37], which classifies six levels of participation according to the degree of interaction with others and the activity’s purpose. Responses were rated from 1 (Daily) to 5 (Less than once a month), and these ratings were subsequently recoded to estimate the number of days per week spent on each activity.

Cognitive failures in daily life were measured in March 2021 using the 10-item short form of the Cognitive Failures Questionnaire (CFQ) [38]. The questions addressed common lapses in attention, perception, and memory, such as confusing left and right when giving directions or not noticing desired items in a supermarket (refer to Supplementary Table S1). Participants reported how often they experienced each lapse over the past six months on a 5-point scale (1 = very often, 5 = never). The scores were summed, so lower totals reflected more frequent cognitive failures.

### 2.3. Statistical Analyses

Data analysis was conducted using R software (version 4.4.1, R Foundation). K-means cluster modelling (kml) was used to identify distinct cluster trajectories of total ADL and total IADL scores. The kml algorithm (R package kml) is a non-parametric, iterative hill-climbing method that makes no assumptions about the shape of the trajectories [39]. The analysis was specified to allow between 2 and 6 clusters (trajectories), and for each specified number of clusters, the algorithm was run with 1000 different random starts to ensure stability. The optimal number of clusters was identified by maximising the Genolini variant of the Calinski–Harabasz (CH) index [21]. This metric assesses partition quality by calculating the ratio of between-cluster variance to within-cluster variance; a higher value indicates greater cluster separation and compactness. In addition to the CH index, we evaluated the interpretability of the resulting patterns to ensure the identified trajectories were distinct and non-redundant. Baseline comparisons of demographics and scores (ADL, IADL, depression, social support, social isolation, social engagement, CFQ) across clusters were conducted using the Kruskal–Wallis test with post hoc pairwise comparisons (Dunn) using Bonferroni adjustment and standardised mean differences (Hedges’ g) (see Supplementary Table S5) for continuous variables and Pearson’s Chi-squared or Fisher’s exact tests for categorical variables, as appropriate.

This study employed linear mixed-effect models to investigate the associations between ADL/IADL clusters (categorised as Stable, Medium, and High) and both ADL/IADL scores and depression scores over time. All models incorporated random effects for respondents to account for individual variability, as well as fixed effects for the ADL/IADL clusters, time, and their interactions. Additionally, the models were adjusted for relevant covariates to enhance the robustness of the findings. The model estimates were obtained using the maximum likelihood method. Lastly, this study employed time-to-event analysis to quantify the risk of increases in depression (≥5-point increases in the total 8-item CES-D score) by cluster. We plotted Kaplan–Meier survival curves for remaining free of a ≥5-point increase in CES-D score, stratified by cluster, and compared the clusters using log-rank tests. We then fitted Cox proportional hazards models for the ≥5-point outcome, with cluster membership as the main predictor. To account for participants with severe depressive symptoms at baseline, the baseline total depression score was included as a covariate in all Cox models. Three Cox models were run: (i) adjusted for demographics, baseline total depression scores and chronic disease count; (ii) additionally adjusted for social support, isolation, and engagement; (iii) additionally adjusted for CFQ scores. The proportional hazards assumption was assessed for the fully adjusted Cox models (Model 3) using scaled Schoenfeld residual tests. No evidence of non-proportional hazards was detected for trajectory-group membership or other covariates (see Supplementary Table S6). Accordingly, time-varying coefficients were not required in the models. Hence, we report hazard ratios (HRs) with 95% confidence intervals for each cluster (Medium and High) relative to the Stable cluster. To aid interpretation, we also derived median time-to-event estimates from Kaplan–Meier curves within each cluster when appropriate.

## 3. Results

### 3.1. Identification of Functional Trajectories

For total ADL scores, the Genolini variant of the Calinski–Harabasz (CH) index reached its maximum value at three clusters, indicating this was the optimal solution (see Supplementary Table S7). For total IADL scores, the CH index suggested a four-cluster solution was optimal. However, upon inspection, the fourth cluster did not represent a meaningfully distinct trajectory; it essentially mirrored the stable trajectory of the lowest group, differing only by a marginally higher initial score (see Supplementary Figure S2). Including this fourth cluster would have yielded two redundant “stable” trajectories. Hence, selecting three clusters effectively collapses two stable-like subgroups and is unlikely to change the main contrasts (Medium/High vs. Stable), although any subtle within-stable differences could attenuate estimates toward the null. Moreover, to ensure parsimony and interpretability of the resulting patterns, we selected the three-cluster solution for IADL. Thus, the final analysis proceeded with three distinct trajectory groups for both ADL and IADL domains.

### 3.2. Trajectory Clusters of Functional Difficulty

**Figure 1:**
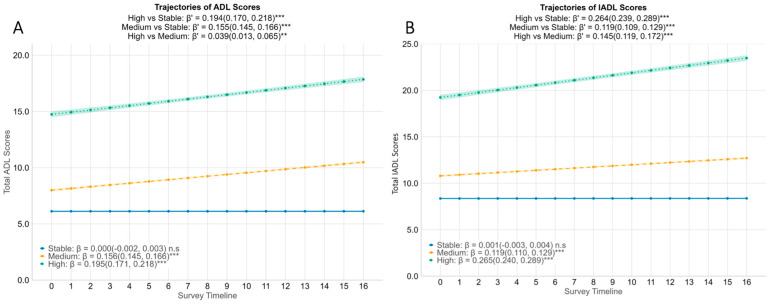
Trajectories of functional difficulty by cluster group. Estimated mean ADL difficulty score (**A**) and IADL difficulty score (**B**) with 95% CI are stratified by functional trajectory clusters identified via kml (blue = Stable, yellow = Medium increase in functional difficulty, green = High increase in functional difficulty). β coefficients with 95% CIs are derived from linear mixed-effect models adjusted for age, gender, education, housing, marital status, number of chronic diseases, social support, isolation and engagement, and cognitive failures scores. Interpretation: Within each cluster, β represents the increase in functional difficulty over time for either ADL or IADL. Between clusters, β′ represents the difference in the rate of change in ADL or IADL scores between the High/Medium cluster and the Stable cluster. ** *p* < 0.01, *** *p* < 0.001, n.s: not significant. ADLs = Activities of Daily Living, IADLs = Instrumental Activities of Daily Living, CI = Confidence Interval.

While overall ADL and IADL scores increased during the follow-up period (see Supplementary Figure S3), we identified three clear patterns of functional change over a 4-year follow-up using k-means longitudinal clustering. We labelled the clusters Stable, Medium (medium increase in functional difficulties over time), and High (the highest increase in functional difficulties over time) based on their patterns (Table 1). The Stable cluster comprised respondents (N = 4017 for ADL and N = 3637 for IADL), who maintained consistently low functional difficulty scores across the entire timeline. The Medium cluster (N = 214 for ADL and N = 556 for IADL) comprised participants with moderate functional difficulty who gradually increased over time. Finally, the High cluster had the fewest participants (N = 42 in ADL; N = 80 in IADL), was characterised by greater initial difficulty, and showed a more rapid increase in functional difficulty. Figure 1 shows the mean trajectories of each cluster.

For ADL (Figure 1A), the stable group’s score remained unchanged (β = 0.000, 95% CI = −0.002 to 0.003), while the Medium group started slightly higher and increased steadily in the ADL difficulty score (β = 0.156, 95% CI = 0.145 to 0.166). The High ADL cluster had the highest baseline functional difficulty score (median score of 15, Table 1) and showed the steepest rise (β = 0.195, 95% CI = 0.171 to 0.218). Between-cluster differences in slope were significant: relative to the stable trajectory, both the Medium and High groups showed a significantly faster increase in ADL difficulty scores across timepoints (Medium vs. Stable: β′ = 0.155, 95% CI = 0.145 to 0.166; High vs. Stable: β′ = 0.194, 95% CI = 0.170 to 0.218). Even between the High versus Medium increase in ADL difficulty clusters, there was a modestly faster increase in ADL difficulty scores in the High group (β′ = 0.039, 95% CI = 0.013 to 0.065), indicating a significantly accelerated increase in the ADL difficulty for the small subset of respondents. A parallel pattern was observed in IADL scores (Figure 1B): the Stable cluster remained unchanged over time, the Medium cluster increased by 0.119 points quarterly, and the High cluster rose by 0.265 points quarterly. In terms of differences in the rate of increase between clusters, both the Medium and High groups showed a significantly faster increase in IADL difficulty scores compared to the Stable group (Medium vs. Stable: β′ = 0.119, 95% CI = 0.109 to 0.129; High vs. Stable: β′ = 0.264, 95% CI = 0.239 to 0.289). In summary, the k-means clustering model provided clusters that represented divergent functional ageing paths: one essentially stable, one with a mild increase in functional difficulties, and one with a severe increase in functional difficulties over the four years.

### 3.3. Sociodemographic and Baseline Characteristics of Trajectory Groups

Participants in the Medium and High clusters differed significantly from those in the Stable cluster across a range of baseline characteristics, as shown in Table 1. Baseline age increased across trajectory severity; for ADL clusters, median age (Q1–Q3) was 63 (59–68) in Stable, 66 (62–72) in Medium, and 70 (62–74) in High, with a similar pattern for IADL clusters (*p* < 0.001; Table 1). The Medium and High ADL and IADL clusters had more chronic diseases (*p* < 0.001) compared to their respective stable groups. Women were more common in the Medium and High IADL clusters (*p* < 0.001), while gender distribution was similar among the ADL clusters. Regarding marital status, in the IADL clusters, participants who were separated, divorced, or widowed were disproportionately represented in the Medium/High clusters (*p* < 0.001). Lower educational levels and smaller HDB housing types were more prevalent in the Medium/High clusters, whereas university education and private housing were predominantly found in the Stable cluster (*p* < 0.001). Baseline total ADL and IADL difficulty scores were, as expected, significantly higher in the Medium and High clusters for the respective domains (*p* < 0.001). Psychosocial measures at baseline reflected the same pattern: higher baseline depression (e.g., medians of baseline total depression score increased from 19 in Stable group to 25/29 (ADL) and 22/27 (IADL) in the Medium/High groups) and social isolation scores, along with lowered social support and social engagement, characterised the Medium/High clusters in both domains (*p* < 0.001). Furthermore, Medium and High clusters also reported more frequent cognitive failures (i.e., lower total CFQ scores indicate more frequent failures). Collectively, these patterns reveal a consistent pattern of socioeconomic, psychosocial, and cognitive disadvantages among participants with higher initial ADL and IADL difficulty levels.

### 3.4. Depression Trajectories by Functional Clusters

**Figure 2:**
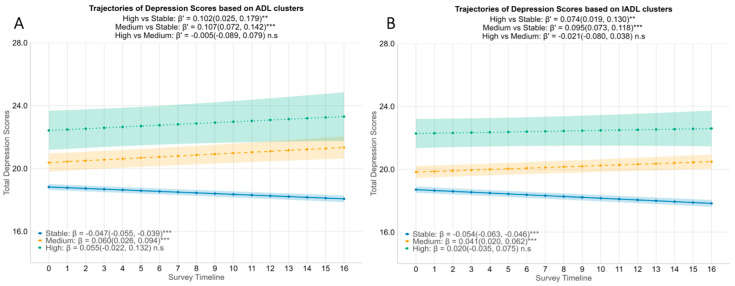
Associations between functional difficulty trajectory clusters and depression scores of the participants. Estimated mean depression scores by ADL difficulty clusters (**A**) and IADL difficulty clusters (**B**) with 95% CI are stratified by functional trajectory clusters identified via kml (blue= Stable, yellow = Medium increase in functional difficulty, green = High increase in functional difficulty). β coefficients with 95% CIs are derived from linear mixed-effect models adjusted for age, gender, education, housing, marital status, number of chronic diseases, social support, isolation and engagement, and cognitive failures scores. Interpretation: Within each cluster, β represents the increase in depression scores over time for either ADL clusters or IADL clusters. Between clusters, β′ represents the difference in the rate of change in depression scores between the High/Medium cluster and the Stable cluster. ** *p* < 0.01, *** *p* < 0.001, n.s: not significant. ADLs = Activities of Daily Living, IADLs = Instrumental Activities of Daily Living, CI = Confidence Interval.

While aggregate depression scores decreased over four years (see Supplementary Figure S4), stratifying by functional difficulty clusters revealed distinct symptom trajectories (Figure 2). The Stable ADL group exhibited a gradual improvement, with 8-item CES-D scores decreasing significantly by 0.047 points per quarter (95% CI = −0.055 to −0.039). In contrast, the Medium ADL cluster experienced a significant rise in depression scores (β = 0.060, 95% CI = 0.026 to 0.094). The High ADL cluster displayed a similar upward trend, though this increase did not reach statistical significance (β = 0.055, 95% CI = −0.022 to 0.132). When compared to the Stable group, both the Medium and High groups showed significantly faster increases in depression scores; the difference in slope was marked for both Medium (β′ = 0.107, 95% CI = 0.072 to 0.142) and the High (β′ = 0.102, 95% CI = 0.025 to 0.179) clusters.

IADL clusters mirrored these patterns (Figure 2B). Depression scores in the Stable IADL group improved over time (β = −0.054, 95% CI = −0.063 to −0.046). Opposing this trend, the Medium and High IADL clusters experienced an upward trend in depression scores, although the increase in the High IADL group was not statistically significant (Medium: β = 0.041, 95% CI = 0.020 to 0.062; High: β = 0.020, 95% CI = −0.035 to 0.075). Nonetheless, differences between groups were apparent, with both the Medium and High IADL groups showing a significantly faster increase in depression scores compared to the Stable group (Medium vs. Stable: β′ = 0.095, 95% CI = 0.073 to 0.118; High vs. Stable: β′ = 0.074, 95% CI = 0.019 to 0.130). Collectively, these findings demonstrate that older adults with moderate to high functional difficulties follow a diverging mental health path compared to their peers with stable function. While the functionally stable group saw symptom relief over time, those with moderate or high ADL/IADL difficulties experienced a progressive increase in depression.

### 3.5. Incidence of Depression by Functional Trajectory Group

**Figure 3:**
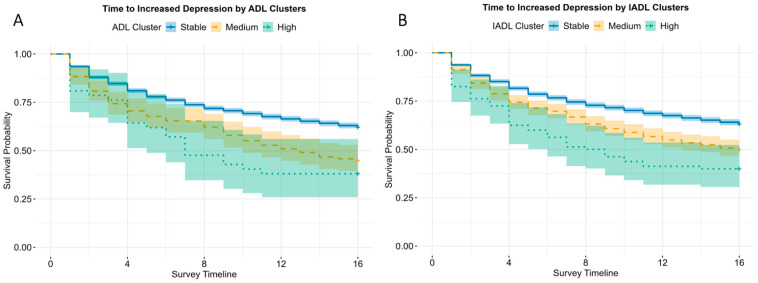
Kaplan–Meier curves for time to increased depression (first ≥5-point rise in 8-item CES-D scores from baseline) by (**A**) ADL and (**B**) IADL trajectory clusters. The Stable cluster (blue) maintained the highest probability of remaining event-free, while Medium (yellow) and High (green) clusters showed earlier and more frequent events. Median time-to-event was not reached in the Stable cluster and occurred earlier in the Medium and High clusters (ADL: 3.25 years and 1.75 years; IADL: 4.0 years and 2.1 years).

Time-to-event analyses showed that the incidence of increased depression (an ≥5-point increase in CES-D scores) occurred more frequently and earlier among participants in the High functional difficulty group (Figure 3; Table 2). Overall, 39.1% of the participants experienced increased depression over follow-up. Log-rank tests demonstrated significant differences in time-to-event (i.e., increased depression) across clusters for both ADL and IADL (*p* < 0.001). For ADL, the median time-to-increased depression was not estimable in the Stable group (event rate 38.0%), whereas the median time was 3.25 years for the Medium group, and 1.75 years for the High group, with progressively higher event rates across clusters (Stable: 38.0%, Medium: 55.1%, High: 61.9%). In multivariable Cox models adjusted for demographics and baseline total depression scores (Model 1), hazards were elevated in a graded manner (Medium: HR = 1.80, 95% CI = 1.48 to 2.19; High: HR = 2.51, 95% CI = 1.67 to 3.75), and remained elevated for additional adjustment for social and cognitive factors (Model 3: Medium HR = 1.71, 95% CI = 1.41 to 2.09; High: HR = 2.37, 95% CI = 1.58 to 3.55; see Supplementary Table S8). These findings indicate that participants in the Medium group had about 1.7 times the hazard of a 5-point increase in CES-D score during follow-up compared with the Stable group, whereas the High group had about twice the hazard.

For IADL, the median time-to-increased depression was not reached in the Stable group (event rate 36.9%), whereas the median time-to-event was 4.0 years in the Medium group, and 2.1 years in the High group. The percentage of respondents with increased depression scores rose from 36.9% (Stable) to 50.2% (Medium) and 60.0% (High). In demographic and baseline total depression score-adjusted analyses (Model 1), both the Medium (HR = 1.64, 95% CI = 1.42 to 1.90) and High (HR = 2.34, 95% CI = 1.72 to 3.19) groups had higher hazards ratios of increased depression than the Stable group. After further adjustment for social factors and cognition (Model 3, see Supplementary Table S8), hazards remained elevated (Medium HR = 1.60, 95% CI = 1.38 to 1.85; High: HR = 2.20, 95% CI = 1.61 to 3.01). Overall, poorer functional trajectories were associated with earlier and more frequent increased depression; however, estimates for the smallest “High” clusters should be interpreted with greater caution, given reduced precision.

To assess robustness given the small sample size of the High clusters, we conducted sensitivity analyses using a stricter event definition (CES-D increase of ≥10 points) and Firth penalised Cox regression. Under the ≥10-point definition, associations were generally in the same direction, but confidence intervals widened (e.g., fully adjusted ADL High: HR = 5.65, 95% CI = 3.14 to 10.14; fully adjusted IADL High: HR = 4.49, 95% CI = 2.78 to 7.25). Firth penalisation further shrank estimates toward the null, especially for the smallest High clusters, highlighting limited power for those subgroup contrasts. These sensitivity results are reported in Supplementary Tables S2–S4.

## 4. Discussion

This longitudinal study demonstrates that patterns of functional change, not only baseline function, identify community-dwelling older adults at elevated risk of increased depression. Across four years of quarterly follow-up, we identified three trajectory groups in both ADL and IADL domains: Stable function, Medium increase, and High increase in difficulties. Memberships in the Medium and High groups were associated with a significantly higher hazard of increased depression, even after adjustment for baseline sociodemographic, chronic diseases, baseline depression, psychosocial and cognitive factors. Importantly, the timing of increased depression differed by trajectory severity, with earlier median time-to-event in the more adverse trajectories, while the Stable group did not reach the median within follow-up. This emphasises a dynamic link between physical and mental health in older adults.

Previous research has demonstrated an association between functional difficulties and depression. However, our study extends this literature by explicitly accounting for unobserved heterogeneity in late-life disability progression using high-frequency assessments [17,40,41]. While the majority experienced minimal functional change and relatively stable depressive symptoms, a smaller subgroup exhibited accelerated functional difficulty accompanied by greater mental health burden. In fully adjusted Cox models, membership in more adverse functional-trajectory groups was associated with substantially higher hazards of increased depression compared to the Stable group (ADL: Medium HR = 1.71; High HR = 2.37; IADL: Medium HR = 1.60; High HR = 2.20). Compared with studies using less frequent measurements, our hazard ratios are generally larger than those reported for baseline disability scores predicting incident depressive symptoms in Scientific Reports (CHARLS 2015–2018: HR = 1.090, 95% CI 1.058–1.123), though direct comparison is limited because the study modelled baseline ADL disability as a continuous score rather than trajectory membership [42]. In contrast, the LUCAS study, with six biannual observations over 10 years, reported similar in magnitude to hazards between functional decline/frailty and depressed mood (e.g., functional decline → depressed mood HR = 2.32, 95% CI 1.703–3.172) [43]. Importantly, our quarterly design additionally revealed distinct trajectory shapes (Stable, Medium and High groups) and enabled estimation of time windows for increases in depression, which may be obscured when functional change is averaged over long intervals.

Using Kaplan–Meier methods, we estimated the time to a ≥5-point increase in depressive symptoms across functional-difficulty clusters. Median time-to-event occurred earlier in more adverse functional trajectories (ADL: 3.25 years in the Medium; 1.75 years in the High; Stable not reached; IADL: 4.0 years in the Medium; 2.1 years in the High; Stable not reached). Although these estimates are unadjusted, they align with the covariate-adjusted Cox models, indicating that functional trajectory membership remains informative even after accounting for baseline demographic, socioeconomic, baseline depression, psychosocial, and cognitive differences. This pattern is consistent with evidence that functional difficulty and depression are tightly linked over shorter time scales. Chang et al. (2009) reported a non-significant trend towards a higher risk of depression at the time of increased functional difficulty over the subsequent six months [44]. Furthermore, yearly panel data indicated a strong contemporaneous effect of disability change on depressive symptoms, with a weaker reverse lag, supporting functional difficulty as a leading indicator [6]. Taken together, our findings provide novel evidence by integrating data-driven functional categories with empirically derived onset windows for the increase in depressive symptoms. This dual focus offers better predictions of increased depression compared to traditional models, which may obscure class heterogeneity, thereby reducing the precision of rate-of-change estimates for health outcomes [18,19,20].

Collectively, declining physical function may restrict social activities and participation, resulting in social isolation, which may contribute to depression in later life [24]. In our cohort, those in the Medium and High clusters were more likely to be socially disadvantaged (i.e., lower education, widowed, older, etc.), potentially amplifying vulnerability as functional difficulties emerge. This pattern aligns with the social determinants of health framework, which posits that social disadvantage can shape mental health by reducing social resources and increasing isolation risk [45]. At the same time, the same pathways may also be protective: maintaining social participation, preserving meaningful social roles and sustaining perceived social support may buffer the psychological impact of increased functional disability and attenuate progression to increased depression [17,25,26].

Beyond social pathways, functional deterioration may contribute to increased depression through several interrelated psychological and behavioural mechanisms. First, increasing ADL/IADL difficulty can erode independence and perceived control, as individuals become less able to manage personal care, household tasks, and daily logistics without assistance. Loss of mastery and autonomy is a recognised psychosocial mechanism through which functional limitations may translate into poorer mental health [46]. Second, functional difficulties can trigger activity restriction and role loss (e.g., reduced participation in family roles, volunteering, community activities), diminishing access to rewarding, valued experiences and increasing feelings of helplessness or demoralisation [47]. Third, the day-to-day burden of disability may generate chronic stress through heightened effort, frustration, and increased dependence on others, which can cumulatively increase vulnerability to depressive symptoms [48]. These pathways align with the disablement process frameworks that conceptualise disability as the downstream consequence of functional limitations interacting with environmental demands, and they offer plausible mechanisms linking functional trajectories to subsequent mood deterioration. Conversely, preserving a sense of control, autonomy, and mastery despite functional limitations may help buffer against increased depression, even when functional impairment progresses. Together, these psychological and behavioural pathways suggest that the impact of disability on depression may depend in part on the resources available to maintain connection, agency and meaning.

An important alternative is that early depressive symptoms may shape how functional difficulty is experienced and reported. Early and subclinical depressive symptoms could contribute to higher self-reported functional difficulty through reduced motivation and energy, psychomotor slowing, and negatively biased appraisals of effort (i.e., everyday tasks feeling harder even before objective disability progresses) [49]. Prior longitudinal research supports bidirectional associations between depressive symptoms and functional disability in older adults, underscoring that the temporal ordering may not be strictly unidirectional [42,50]. Accordingly, we frame functional trajectory membership as a useful risk marker and early warning signal for increased depression rather than definitive evidence of a unidirectional causal pathway. Future work using reciprocal time-varying or cross-lagged models could better disentangle within-person dynamics over shorter time scales.

These findings have important implications for public health and clinical practice by identifying risk-stratified windows for monitoring and intervention. Trajectory membership can guide the frequency of functional and mood screening: individuals in the Stable trajectory may require routine, lower-intensity check-ins, whereas those in the Medium trajectory may benefit from at least annual review to detect emerging risk. By contrast, the High groups experienced shorter time-to-increased depression, suggesting more frequent (e.g., quarterly) check-ins and timely targeted support once rapid deterioration is detected. Interventions can likewise be tiered to severity: prevention-focused strategies that sustain physical activity and social participation may be most appropriate for Stable trajectories; early rehabilitation and functional support (e.g., occupational therapy to maintain IADL capacity, and home/environmental modifications) may be prioritised for Medium trajectories [51,52]; and proactive case management with integrated care (including timely geriatric assessment, caregiver engagement, and linkage to psychological care when indicated) may be warranted for High trajectories [53]. Although quarterly assessment may not be feasible as a universal, clinic-based requirement, a tiered approach is feasible: brief ADL/IADL self-report tools can be embedded in community follow-ups for higher-risk subgroups via telephone or online survey platforms, with resource-intensive multidisciplinary assessment reserved for those with rapid decline. This approach supports efficient resource allocation while maintaining preventive monitoring in the majority with stable function.

Policy responses must also address inequalities in vulnerability and access. Our study found that older adults of lower socioeconomic status (i.e., lower educational attainment, residing in smaller housing) are overrepresented in high functional-difficulty trajectories and exhibit higher baseline depressive symptoms. Evidently, functional difficulties carry disproportionate mental health consequences for these disadvantaged groups. Outreach efforts should therefore prioritise these populations by ensuring equitable access to subsidised home care, mental health services, and chronic disease management [52,53]. Disability prevention strategies should move beyond physical independence alone and incorporate mental health promotion, but these strategies ought to be tailored to meet the specific needs of lower socio-economic strata of older adults.

This study’s strengths include its high-frequency longitudinal design and large sample size, which enabled the detection of fine-grained changes via robust clustering. The use of seventeen time-points over four years enhanced confidence in trajectory classifications compared to studies with fewer observation points. Furthermore, examining both ADLs and IADLs revealed similar patterns, strengthening the conclusion that functional difficulty (whether basic or instrumental) is detrimental to mental health [12]. By employing mixed models and survival analysis with covariate adjustment, we were able to isolate the effects of functional trajectories while controlling for confounders.

Several limitations merit consideration. First, the cohort comprised community-dwelling older adults in Singapore and was predominantly younger-old at baseline, which may limit generalisability to older and more clinically frail populations. We restricted analyses to respondents with complete longitudinal functional and depression data to ensure consistent trajectory estimation across individuals. As a result, clusters were defined within a complete-case analytic sample, and attrition and mortality over four years of quarterly follow-up may have disproportionately removed individuals with the steepest health declines. This restriction may over-represent healthier participants, and bias associations towards the null; thus, associations between functional deterioration and increased depression may be conservative and most applicable to community-dwelling older adults with sufficient health and functional capacity to sustain longitudinal participation. Institutionalised populations are not represented in this sampling frame. Residual confounding by comorbid health conditions may influence time-to-increased depression. Second, inference for the smallest trajectory groups is constrained by limited precision. The High clusters were small (High ADL, n = 42; High IADL, n = 80), resulting in wider confidence intervals in fully adjusted Cox models. Sensitivity analyses using an alternative event definition (≥10-point CES-D increase) and Firth penalised Cox regression yielded more conservative estimates, indicating that hazard-ratio magnitudes for the High clusters should be interpreted cautiously. Third, ADL and IADL difficulties were self-reported. Although frequent measurements improve temporal resolution, reporting bias remains a concern; future work could integrate wearable activity data to validate self-reports and enhance accuracy. Fourth, trajectory solutions may be sensitive to modelling choices (e.g., selecting three clusters for IADL despite a Calinski–Harabasz score favouring four). Replicating in larger samples and sensitivity analyses using alternative longitudinal clustering approaches (such as shape-aware dynamic time warping using the dtwclust package [54]) would strengthen robustness. Finally, context may constrain the generalisability. Singapore is a dense, city-state with broad access to subsidised primary and outpatient care (e.g., Community Health Assist Scheme and Healthier SG) and an expanding community ageing ecosystem (e.g., Active Ageing Centres), which may facilitate earlier identification of decline, linkage to support, and maintenance of community participation. These structural features and culturally shaped caregiving norms may moderate how functional difficulty translates into depressive symptoms. Hence, the observed trajectory structure and effect sizes may differ in settings with weaker primary care/community supports or different family support norms. Conversely, trajectories may also differ in Western settings, where living arrangements, institutionalisation patterns, and cultural thresholds for reporting difficulties vary. In addition, portions of follow-up overlapped with the COVID-19 period, which may have influenced functional activity and mood through mobility restrictions and altered social contact. Replication across diverse health systems and post-pandemic contexts is warranted.

## 5. Conclusions

In a rapidly ageing society, promoting healthy ageing requires addressing both physical and mental dimensions. This study demonstrates that older adults followed distinct ADL and IADL trajectory pathways, and that those on trajectories of greater functional difficulty face a significantly higher risk of worsening depression within about one to two-year period. By using a novel clustering approach, we identified high-risk groups without imposing preconceived model shapes, providing a data-driven perspective on how functional difficulty unfolds in older adults. Preventing depression in later life may therefore require a dual focus on mental health and functional ability. Early rehabilitation and supportive services for individuals with accelerating ADL and IADL impairments represent a viable strategy to reduce the burden of late-life depression. However, given the shrinking family structures and the declining availability of home-based caregivers, relying solely on traditional family support is increasingly unsustainable. Consequently, there is an urgent need to leverage technologies, ranging from smart home automation and digital assistive tools that support daily tasks to wearable sensors for early detection, to help older adults compensate for functional deficits and bridge the care gap. Ultimately, recognising and addressing emerging functional difficulties is not only vital for preserving independence but serves as a key component of depression prevention in ageing societies.

## Figures and Tables

**Table 1:** Demographic characteristics of ADL and IADL clusters at baseline.

Characteristic	ADL Cluster			*p*-Value ^2^	IADL Cluster			*p*-Value ^2^
	Stable	Medium	High		Stable	Medium	High	
	N = 4017 ^1^	N = 214 ^1^	N = 42 ^1^		N = 3637 ^1^	N = 556 ^1^	N = 80 ^1^	
Baseline Age	63 (59, 68)	66 (62, 72)^α^	70 (62, 74) ^α^	<0.001	63 (59, 67)	68 (63, 72) ^α^	71 (65, 75) ^α,†^	<0.001
Gender				0.909				<0.001
Male	1919 (94.2%)	100 (4.9%)	19 (0.9%)		1791 (87.9%)	214 (10.5%)	33 (1.6%)	
Female	2098 (93.9%)	114 (5.1%)	23 (1.0%)		1846 (82.6%)	342 (15.3%)	47 (2.1%)	
Marital Status				0.081				<0.001
Married	3234 (94.3%)	164 (4.8%)	33 (1.0%)		2965 (86.4%)	412 (12.0%)	54 (1.6%)	
Single	370 (95.1%)	15 (3.9%)	4 (1.0%)		343 (88.2%)	37 (9.5%)	9 (2.3%)	
Separated/Divorced/Widowed	413 (91.2%)	35 (7.7%)	5 (1.1%)		329 (72.6%)	107 (23.6%)	17 (3.8%)	
Education				<0.001				<0.001
No/Primary	1213 (88.9%)	124 (9.1%)	27 (2.0%)		932 (68.3%)	374 (27.4%)	58 (4.3%)	
Secondary	1116 (96.0%)	41 (3.5%)	6 (0.5%)		1055 (90.7%)	98 (8.4%)	10 (0.9%)	
Post-Secondary	944 (95.9%)	36 (3.7%)	4 (0.4%)		912 (92.7%)	65 (6.6%)	7 (0.7%)	
University	744 (97.6%)	13 (1.7%)	5 (0.7%)		738 (96.9%)	19 (2.5%)	5 (0.7%)	
Housing				<0.001				<0.001
1–3 room HDB	689 (89.2%)	65 (8.4%)	18 (2.3%)		569 (73.7%)	170 (22.0%)	33 (4.3%)	
4–5 room HDB	2406 (94.3%)	128 (5.0%)	18 (0.7%)		2182 (85.5%)	333 (13.0%)	37 (1.4%)	
Private Housing	922 (97.2%)	21 (2.2%)	6 (0.6%)		886 (93.4%)	53 (5.6%)	10 (1.1%)	
Number of Chronic Diseases	1 (0, 2)	2 (1, 3) ^α^	3 (2, 4) ^α,†^	<0.001	1 (0, 2)	2 (1, 3) ^α^	3 (2, 4) ^α,†^	<0.001
Baseline ADL Scores	6 (6, 6)	7 (6, 10) ^α^	15 (12, 18) ^α,†^	<0.001	6 (6, 6)	6 (6, 7) ^α^	11 (6, 15) ^α,†^	<0.001
Baseline IADL Scores	8 (8, 9)	11 (8, 15) ^α^	23 (18, 27) ^α,†^	<0.001	8 (8, 8)	11 (9, 12) ^α^	19 (15, 24) ^α,†^	<0.001
Baseline Total Depression Scores	19.0 (15.0, 23.0)	25.0 (20.0, 29.0) ^α^	29.0 (25.0, 33.0) ^α,†^	<0.001	19.0 (15.0, 23.0)	22.0 (18.0, 27.0) ^α^	27.0 (23.0, 32.0) ^α,†^	<0.001
Baseline Social Support Scores	26.0 (21.0, 29.0)	21.0 (18.0, 27.0) ^α^	22.0 (17.0, 28.0) ^α^	<0.001	26.0 (21.0, 29.0)	23.0 (20.0, 28.0) ^α^	23.0 (17.0, 28.0) ^α^	<0.001
Baseline Social Isolation Scores	2.0 (1.0, 3.0)	3.0 (2.0, 3.0) ^α^	3.0 (3.0, 4.0) ^α,†^	<0.001	2.0 (1.0, 3.0)	2.0 (2.0, 3.0) ^α^	3.0 (2.0, 4.0) ^α,†^	<0.001
Baseline Social Engagement Scores	0.9 (0.3, 1.6)	0.5 (0.1, 1.0) ^α^	0.1 (0.0, 1.0) ^α^	<0.001	0.9 (0.3, 1.6)	0.6 (0.1, 1.3) ^α^	0.1 (0.0, 0.9) ^α,†^	<0.001
Baseline Total CFQ Scores	38.0 (33.0, 42.0)	33.0 (30.0, 39.0) ^α^	30.0 (23.0, 37.0) ^α^	<0.001	38.0 (33.0, 42.0)	35.0 (30.0, 40.0) ^α^	30.0 (24.0, 39.0) ^α,†^	<0.001

^1^ Median (Q1, Q3); n (%), ^2^ Kruskal–Wallis rank sum test with post hoc pairwise comparisons (Dunn) using Bonferroni adjustment; Pearson’s Chi-squared test; Fisher’s exact test. ^α^ Significantly different from Stable cluster, ^†^ Significantly different from Medium clusters. ADLs = Activities of Daily Living, CFQ = Cognitive Failures Questionnaire, HDB = Housing & Development Board, IADLs = Instrumental Activities of Daily Living.

**Table 2:** Association of functional trajectory clusters with hazard of increased depression (Cox proportional hazards regression). Values represent Hazard Ratios (HR) and 95% CI for experiencing a (≥5-point) increase in total CES-D depression score in those in Medium and High clusters relative to the Stable cluster (reference). Model 1 is adjusted for baseline demographics (age, gender, education, housing, marital status, and number of chronic diseases) and baseline total depression score. Model 2 adds social factors (baseline social support, social isolation and social engagement). Model 3 adds cognitive factors (baseline cognitive failure scores). Event rates (% of participants with outcome within each cluster) and median time-to-event (in waves) are provided for context. Participants in Medium or High clusters showed an increased hazard of depression worsening in all models. ‘-’ indicates the median time-to-event was not reached within the follow-up period. *** *p* < 0.001. ADLs = Activities of Daily Living, IADLs = Instrumental Activities of Daily Living.

		Cluster	Hazard Ratio (95% CI)	Outcome; Overall Event Rate	Log-Rank *p*-Value	Median Time-to-Event (Waves)	Event Rate	C-Index (SE)	AIC
ADL	Model 1	Stable	1	Depression increased by 5 points or more; 39.1% event rate	<0.001	-	38.0%	0.61 (0.007)	26,933.70
		Medium	1.80 (1.48, 2.19) ***			13 (3.25 years)	55.1%		
		High	2.51 (1.67, 3.75) ***			7 (1.75 years)	61.9%		
	Model 2	Stable	1					0.63 (0.007)	26,840.40
		Medium	1.75 (1.43, 2.13) ***						
		High	2.48 (1.65, 3.72) ***						
	Model 3	Stable	1					0.63 (0.007)	26,829.40
		Medium	1.71 (1.41, 2.09) ***						
		High	2.37 (1.58, 3.55) ***						
IADL	Model 1	Stable	1	Depression increased by 5 points or more; 39.1% event rate	<0.001	-	36.9%	0.61 (0.007)	26,919.40
		Medium	1.64 (1.42, 1.90) ***			16 (4.0 years)	50.2%		
		High	2.34 (1.72, 3.19) ***			8.5 (2.1 years)	60.0%		
	Model 2	Stable	1					0.63 (0.007)	26,824.90
		Medium	1.63 (1.41, 1.88) ***						
		High	2.32 (1.70, 3.17) ***						
	Model 3	Stable	1					0.63 (0.007)	26,816.20
		Medium	1.60 (1.38, 1.85) ***						
		High	2.20 (1.61, 3.01) ***						

## Data Availability

As the SLP is an ongoing study, the data are not publicly available and are stored under an independent server managed by SMU. Access may be granted only through specific requests to rosa@smu.edu.sg.

## Supplementary Materials

The following supporting information can be downloaded at: https://www.mdpi.com/article/10.3390/healthcare14050629/s1, Table S1. Measurement items; Table S2. Association of functional trajectory clusters with hazard of increased depression (Cox proportional hazards regression); Table S3. Firth penalized Cox proportional hazards models estimating the association between ADL and IADL trajectory clusters (Stable [reference], Medium, High) and the time to first “increased depression” event, defined as a ≥5-point increase in CES-D score from baseline; Table S4. Firth penalized Cox proportional hazards models estimating the association between ADL and IADL trajectory clusters (Stable [reference], Medium, High) and the time to first “increased depression” event, defined as a ≥10-point increase in CES-D score from baseline; Table S5. Standardised mean differences (SMD; Hedges’ g) for pairwise contrasts in baseline continuous variables across ADLs and IADLs trajectory clusters (Stable, Medium, High); Table S6. Proportional hazards assumption diagnostics for fully adjusted Cox models (Model 3) based on scaled Schoenfeld residual tests; Table S7. Calinski–Harabasz scores for determining optimal cluster count (ADL and IADL trajectories); Table S8. Adjusted Cox proportional hazards Model 3 estimating the association between ADL and IADL trajectory clusters (Stable [reference], Medium, High) and time to first increased depression, defined as a ≥5-point increase in CES-D-8 score from baseline; Figure S1. Participants Flow Diagram; Figure S2. Trajectories of total IADL scores by three clusters and four clusters; Figure S3. Overall trajectories of functional difficulty scores (ADL and IADL); Figure S4. Overall trajectories of depression scores over 17 observation points (2020–2024).

## Abbreviations

The following abbreviations are used in this manuscript:

WiSE	Well-being of Singapore’s Elderly
ADLs	Activities of daily living
IADLs	Instrumental activities of daily living
LCGA	Latent Class Growth Analysis
GBTM	Group-Based Trajectory Models
kml	k-means for longitudinal data
SLP	Singapore Life Panel^®^
CES-D	Centre for Epidemiologic Studies-Depression Scale
CFQ	Cognitive Failures Questionnaire
CH	Calinski–Harabasz
CI	Confidence Interval
HDB	Housing & Development Board
CHARLS	China Health and Retirement Longitudinal Study
LUCAS	Longitudinal Urban Cohort Ageing Study
