# Supplementary material for: High-Frequency Functional Trajectories Predict Depressive Worsening in Singapore’s Community-Dwelling Older Adults
Source: Healthcare (Basel). 2026 Mar 2;14(5):629. doi: 10.3390/healthcare14050629 (PMC12984184; doi:10.3390/healthcare14050629)
Supplement: Supplementary file 1 [file healthcare-14-00629-s001.zip › healthcare-4132794-supplementary.pdf]

Supplementary Materials

Table S1. Measurement items

Categories	Measures	Response options
ADLs (Cronbach's α = 0.954)	Because of a health or memory problem, do you have any difficulty with the following activities?<br>1. Dressing, including putting on shoes and socks<br>2. Walking across a room<br>3. Bathing or showering<br>4. Eating, such as cutting up your food<br>5. Getting in or out of bed<br>6. Using the toilet, including getting up and down	1 Not difficult at all<br>2 Somewhat difficult<br>3 Very difficult<br>4 Unable to perform
IADLs (Cronbach's α = 0.867)	How difficult is it for you to perform each of the following activities by yourself?<br>1. Prepare own meals<br>2. Leave the home to purchase necessary items or medication<br>3. Take care of financial matters e.g., paying utilities (electricity, water)<br>4. Use the phone<br>5. Dust, clean-up and other light housework<br>6. Take public transport to leave home<br>7. Take medication as prescribed<br>8. Use the internet for email or other purpose e.g. making purchases or travel reservations, communicating with relatives & friends or searching for information	1 Not difficult at all<br>2 Somewhat difficult<br>3 Very difficult<br>4 Unable to perform
Depression (Cronbach's α = 0.865)	How often have you felt or behave this way during the last week?<br>1. I felt sad<br>2. I felt happy<br>3. I felt lonely<br>4. I felt depressed<br>5. I felt that everything I did was an effort<br>6. My sleep was restless<br>7. I enjoyed life<br>8. I could not get going	1 None of the time<br>2 A little of the time<br>3 Some of the time<br>4 A good bit of the time<br>5 Most of the time<br>6 All of the time
Perceived Social Support (Cronbach's α = 0.963)	1. Someone you can count on to listen to you when you need to talk.<br>2. Someone to confide in or talk to about yourself or your problems.<br>3. Someone who you can count on for help in a time of need.<br>4. Someone to show you love and affection.<br>5. Someone to help you if you were confined to bed.<br>6. Someone to prepare your meals if you were unable to do it.<br>7. Someone to help you with daily chores if you were sick.	1 None of the time<br>2 A little of the time<br>3 Some of the time<br>4 Most of the time<br>5 All the time
Social Engagement (Cronbach's α = 0.650)	Over the last month, how often did you do each of the following activities?<br>1. Visiting friends or family<br>2. Religious activities: including attending church, mosque, temple or other place of worship<br>3. Group activities: including going to clubs, Community Centres or Senior Activity Centres, playing cards/mahjong, etc.	1 Daily (7)<br>2 Several times per week (3.5)<br>3 Once a week (1)<br>4 1-3 times a month (0.25)<br>5 Never (0)

	4. Physical activities: including exercises, swimming, going for a walk, etc.<br>5. Hobbies: including shopping, gardening, attending courses, arts & crafts, etc.<br>6. Spending time outdoors in parks, green spaces and nature<br>7. Volunteering	
Cognitive Failures Questionnaire (Cronbach's α = 0.916)	1. Do you read something and find you haven't been thinking about it and must read it again?<br>2. Do you feel you confuse right and left when giving directions?<br>3. Do you fail to listen to people's names when you are meeting them?<br>4. Do you fail to see what you want in a supermarket, even though it's there?<br>5. Do you have trouble making up your mind?<br>6. Do you feel you forget appointments?<br>7. Do you forget where you put something like a newspaper or a book?<br>8. Do you feel you forget whether you've turned off a light or locked the door?<br>9. Do you feel you forget people's names?<br>10. Do you feel you can't quite remember something although it's "on the tip of your tongue"?	1 Very Often<br>2 Quite Often<br>3 Occasionally<br>4 Very Rarely<br>5 Never

Table S2. Association of functional trajectory clusters with hazard of increased depression (Cox proportional hazards regression). Values represent Hazard Ratios (HR) and 95% CI for experiencing a (≥10-point) increase in total CES-D depression score in those in Medium and High clusters relative to the Stable cluster (reference). Model 1 is adjusted for baseline demographics (age, gender, education, housing, marital status, and number of chronic diseases) and baseline total depression score. Model 2 adds social factors (baseline social support, social isolation and social engagement). Model 3 adds cognitive factors (baseline cognitive failure scores). Event rates (% of participants with outcome within each cluster) and median time-to-event (in waves) are provided for context. '-' indicates the median time-to-event was not reached within the follow-up period. Participants in Medium or High clusters showed an increased hazard of depression worsening in all models.

		Cluster	Hazard Ratio (95% CI)	Outcome; overall event rate	Log-rank p-value	Median time-to-event (Waves)	Event Rate	C-index (SE)	AIC
ADL	Model 1	Stable	1	Depression increased by 10 points or more; 10.7% event rate	<0.001	-	10.0%	0.66 (0.013)	7462.40
		Medium	2.26 (1.60, 3.19) ***			-	19.2%		
		High	5.58 (3.13, 9.94) ***			-	33.3%		
	Model 2	Stable	1					0.68 (0.013)	7437.90
		Medium	2.23 (1.58, 3.16) ***						
		High	5.63 (3.14, 10.09) ***						
	Model 3	Stable	1					0.68 (0.013)	7439.90
		Medium	2.23 (1.58, 3.16) ***						
		High	5.65 (3.14, 10.14) ***						
IADL	Model 1	Stable	1	Depression increased by 10 points or more; 10.7% event rate	<0.001	-	9.4%	0.67 (0.013)	7458.70
		Medium	1.91 (1.47, 2.49) ***			-	16.5%		
		High	4.25 (2.65, 6.83) ***			-	28.8%		
	Model 2	Stable	1					0.68 (0.013)	7432.50
		Medium	1.92 (1.47, 2.49) ***						
		High	4.45 (2.77, 7.17) ***						
	Model 3	Stable	1					0.68 (0.013)	7434.40
		Medium	1.92 (1.48, 2.50) ***						
		High	4.49 (2.78, 7.25) ***						

*** $p < 0.001$. ADLs = Activities of Daily Living, IADLs = Instrumental Activities of Daily Living.

Table S3. Firth penalized Cox proportional hazards models estimating the association between ADL and IADL trajectory clusters (Stable [reference], Medium, High) and the time to first "increased depression" event, defined as a ≥5-point increase in CES-D score from baseline. Results are presented as hazard ratios (HRs) with 95% confidence intervals for three adjustment sets (Models 1–3). The table also reports the overall event rate, log-rank p-value, and median time-to-event (in waves and approximate years) and cluster-specific event rates. '-' indicates the median time-to-event was not reached within the follow-up period.

		Cluster	Hazard Ratio (95% CI)	Outcome; overall event rate	Log-rank p-value	Median time-to-event (Waves)	Event Rate
ADL	Model 1	Stable	1	Depression increased by 5 points or more; 39.1% event rate	<0.001	-	38.0%
		Medium	1.08 (0.86, 1.33)			13 (3.25 years)	55.1%
		High	1.00 (0.59, 1.58)			7 (1.75 years)	61.9%
	Model 2	Stable	1				
		Medium	1.07 (0.86, 1.33)				
		High	0.99 (0.58, 1.55)				
	Model 3	Stable	1				
		Medium	1.06 (0.85, 1.31)				
		High	0.96 (0.57, 1.51)				
IADL	Model 1	Stable	1	Depression increased by 5 points or more; 39.1% event rate	<0.001	-	36.9%
		Medium	0.99 (0.85, 1.13)			16 (4.0 years)	50.2%
		High	0.85 (0.56, 1.22)			8.5 (2.1 years)	60.0%
	Model 2	Stable	1				
		Medium	1.01 (0.88, 1.17)				
		High	0.81 (0.54, 1.16)				
	Model 3	Stable	1				
		Medium	1.01 (0.88, 1.17)				
		High	0.80 (0.53, 1.15)				

Table S4. Firth penalized Cox proportional hazards models estimating the association between ADL and IADL trajectory clusters (Stable [reference], Medium, High) and the time to first "increased depression" event, defined as a ≥10-point increase in CES-D score from baseline. Results are presented as hazard ratios (HRs) with 95% confidence intervals for three adjustment sets (Models 1–3). The table also reports the overall event rate, log-rank p-value, and median time-to-event (in waves and approximate years) and cluster-specific event rates. '-' indicates the median time-to-event was not reached within the follow-up period.

		Cluster	Hazard Ratio (95% CI)	Outcome; overall event rate	Log-rank p-value	Median time-to-event (Waves)	Event Rate
ADL	Model 1	Stable	1	Depression increased by 10 points or more; 10.7% event rate	<0.001	-	10.0%
		Medium	1.13 (0.75, 1.65)			-	19.2%
		High	0.74 (0.21, 1.82)			-	33.3%
	Model 2	Stable	1				
		Medium	1.17 (0.77, 1.70)				
		High	0.77 (0.22, 1.89)				
	Model 3	Stable	1				
		Medium	1.18 (0.77, 1.71)				
		High	0.77 (0.22, 1.89)				
IADL	Model 1	Stable	1	Depression increased by 10 points or more; 10.7% event rate	<0.001	-	9.4%
		Medium	0.90 (0.67, 1.19)			-	16.5%
		High	0.84 (0.37, 1.60)			-	28.8%
	Model 2	Stable	1				
		Medium	0.90 (0.67, 1.18)				
		High	0.86 (0.38, 1.65)				
	Model 3	Stable	1				
		Medium	0.90 (0.67, 1.18)				
		High	0.86 (0.38, 1.65)				

Table S5. Standardised mean differences (SMD; Hedges' g) for pairwise contrasts in baseline continuous variables across ADLs and IADLs trajectory clusters (Stable, Medium, High). Values are Hedges' g for Stable vs Medium, Stable vs High, and Medium vs High comparisons; Bonferroni-adjusted post-*hoc* *p*-values are indicated by asterisks: * $p < 0.05$, ** $p < 0.01$, *** $p < 0.001$. Negative SMDs indicate lower mean values in the first-listed cluster relative to the second-listed cluster. ADLs = Activities of Daily Living, IADLs = Instrumental Activities of Daily Living, SMD = standardized mean difference, CFQ = Cognitive Failures Questionnaire.

Variables	ADL			IADL		
	SMD Stable vs Medium	SMD Stable vs High	SMD Medium vs High	SMD Stable vs Medium	SMD Stable vs High	SMD Medium vs High
Age	-0.52 ***	-0.88 ***	-0.33	-0.76 ***	-1.21 ***	-0.40 *
Number of Chronic Diseases	-0.84 ***	-1.57 ***	-0.49 *	-0.53 ***	-1.46 ***	-0.72 ***
Baseline ADL Scores	-2.18 ***	-9.66 ***	-2.14 ***	-0.76 ***	-4.93 ***	-1.77 ***
Baseline IADL Scores	-1.98 ***	-8.13 ***	-2.11 ***	-1.82 ***	-7.39 ***	-2.25 ***
Baseline Total Depression Scores	-0.78 ***	-1.60 ***	-0.74 **	-0.50 ***	-1.25 ***	-0.67 ***
Baseline Social Engagement Scores	0.40 ***	0.53 ***	0.16	0.23 ***	0.60 ***	0.40 ***
Baseline Social Support Scores	0.50 ***	0.44 *	-0.06	0.25 ***	0.37 **	0.12
Baseline Social Isolation Scores	-0.53 ***	-1.36 ***	-0.77 **	-0.36 ***	-1.00 ***	-0.59 ***
Baseline Total CFQ scores	0.61 ***	1.10 ***	0.40	0.42 ***	1.08 ***	0.57 **

Table S6. Proportional hazards assumption diagnostics for fully adjusted Cox models (Model 3) based on scaled Schoenfeld residual tests.

	ADL (Model 3)			IADL (Model 3)		
Covariate	df	χ^2	p-value	df	χ^2	p-value
Trajectory group	2	0.40	0.818	2	3.30	0.192
Age	1	1.86	0.172	1	1.91	0.167
Sex	1	0.36	0.547	1	0.35	0.553
Education	3	4.73	0.192	3	4.76	0.190
Chronic diseases	1	2.82	0.093	1	2.81	0.094
Marital Status	2	3.40	0.182	2	3.28	0.194
Housing type	2	1.21	0.545	2	1.24	0.537
Baseline depression	1	3.71	0.054	1	3.37	0.066
Social isolation	1	1.23	0.268	1	1.03	0.310
Social support	1	1.68	0.194	1	1.63	0.201
Social engagement	1	0.47	0.493	1	0.40	0.525
Cognition	1	0.91	0.341	1	1.09	0.296
GLOBAL	17	21.51	0.204	17	25.47	0.085

Table S7. Calinski-Harabasz scores for determining optimal cluster count (ADL and IADL trajectories).

	Numbers of Cluster	Calinski-Harabasz by Genolini
ADL	2	3087.74
	3	**3381.91**
	4	2789.39
	5	2639.77
	6	2508.74
IADL	2	4239.06
	3	5598.65
	4	**5892.29**
	5	4102.02
	6	4728.52

Bolded values represent the maximum scores of Calinski-Harabasz by Genolini criteria, indicating the optimal number of clusters.

Table S8. Adjusted Cox proportional hazards Model 3 estimating the association between ADL and IADL trajectory clusters (Stable [reference], Medium, High) and time to first increased depression, defined as a ≥5-point increase in CES-D-8 score from baseline. Results are represented as hazard ratios (HRs) with 95% confidence intervals for cluster membership and baseline covariates (age, gender, education, number of chronic diseases, marital status, housing, baseline total depression score, social isolation, social support, social engagement, and total CFQ score). $p < 0.05$ *, $p < 0.01$ **, $p < 0.001$ ***.

Model 3	ADLs	IADLs
	Hazard Ratio (95% CI)	Hazard Ratio (95% CI)
Cluster		
Stable	1	1
Medium	1.71 (1.41, 2.09) ***	1.60 (1.38, 1.85) ***
High	2.37 (1.58, 3.55) ***	2.20 (1.61, 3.01) ***
Age	0.99 (0.98, 1.00) **	0.98 (0.97, 0.99) ***
Gender		
Male	1	1
Female	1.08 (0.97, 1.20)	1.05 (0.95, 1.17)
Education		
No/Primary	1	1
Secondary	0.85 (0.75, 0.97) *	0.92 (0.80, 1.04)
Post-Secondary	0.84 (0.73, 0.97) *	0.91 (0.79, 1.04)
University	0.85 (0.73, 1.00)	0.93 (0.78, 1.09)
Number of Chronic Diseases	1.19 (1.14, 1.24) ***	1.19 (1.14, 1.24) ***
Marital Status		
Married	1	1
Single	1.25 (1.06, 1.47) **	1.24 (1.06, 1.46) **
Separated/Divorced/Widowed	0.97 (0.83, 1.14)	0.95 (0.81, 1.11)
Housing		
1-3 room HDB	1	1
4-5 room HDB	0.94 (0.82, 1.07)	0.94 (0.83, 1.07)
Private Housing	0.83 (0.70, 0.98) *	0.84 (0.71, 1.00) *
Baseline Total Depression Scores	0.91 (0.90, 0.92) ***	0.91 (0.90, 0.92) ***
Baseline Social Isolation Scores	1.25 (1.16, 1.34) ***	1.24 (1.15, 1.34) ***
Baseline Social Support Scores	0.97 (0.96, 0.98) ***	0.97 (0.96, 0.98) ***
Baseline Social Engagement Scores	0.92 (0.88, 0.97) **	0.93 (0.88, 0.98) **
Baseline Total CEQ Scores	0.99 (0.98, 0.99) ***	0.99 (0.98, 0.99) **

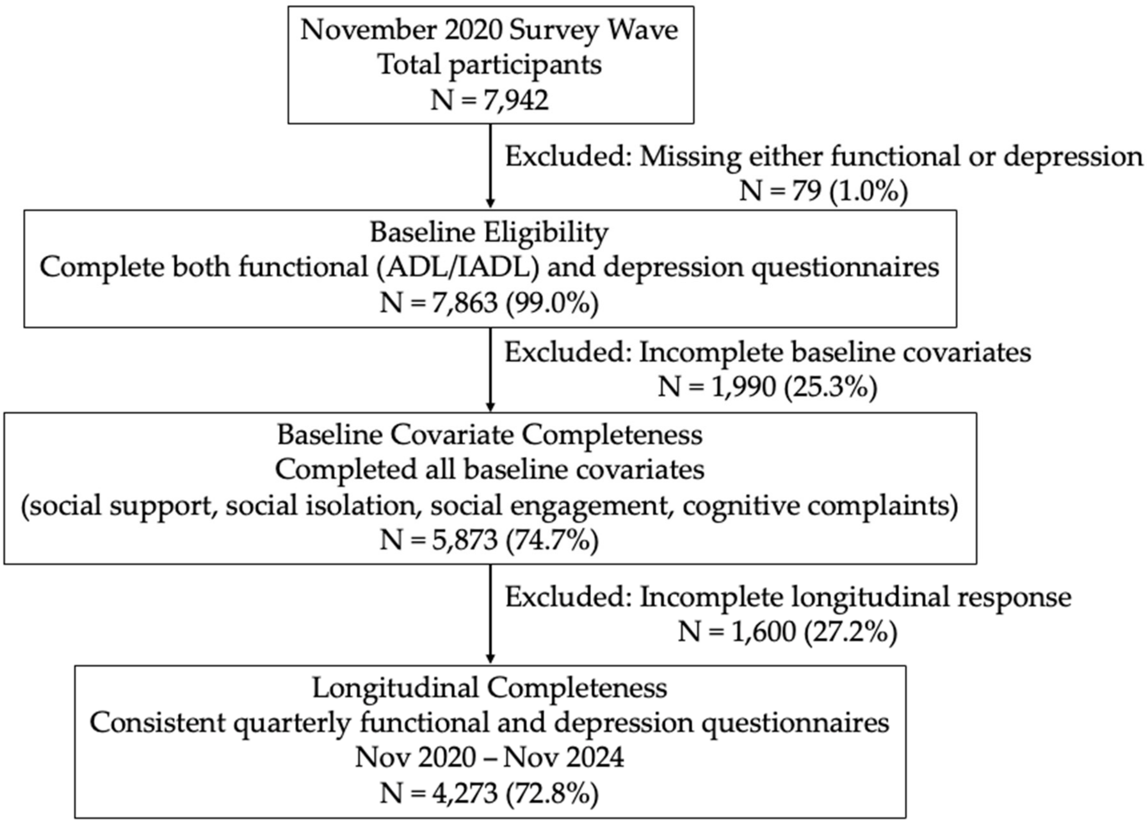

Figure S1. Participants Flow Diagram.

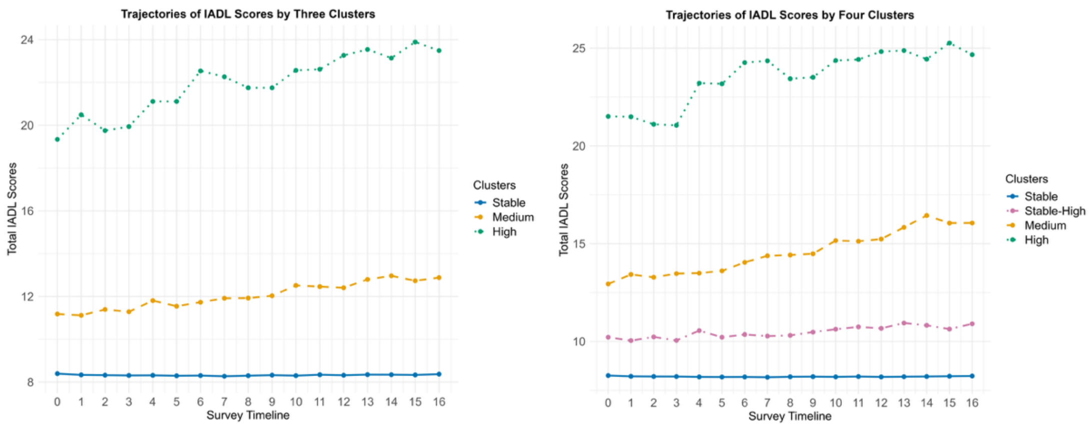

Figure S2. Trajectories of total IADL scores by three clusters and four clusters. IADLs = Instrumental Activities of Daily Living.

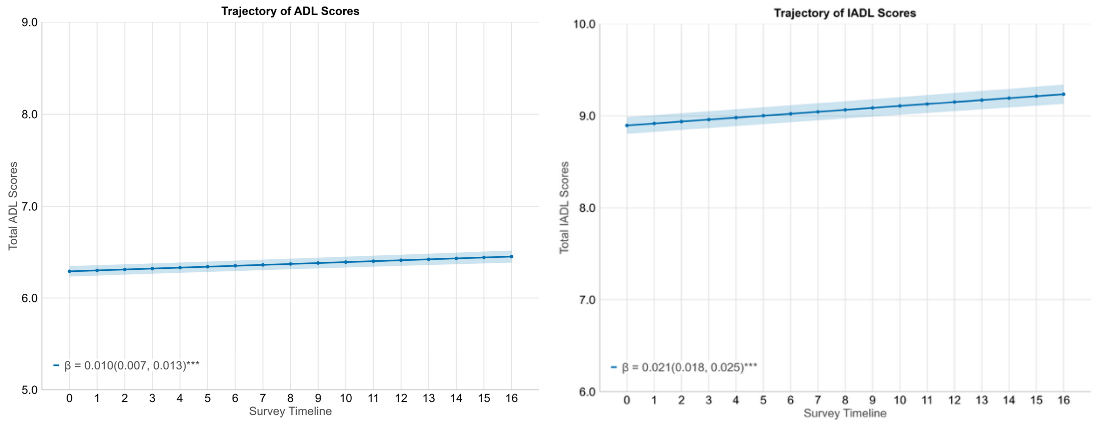

Figure S3. Overall trajectories of functional difficulty scores (ADL and IADL). Plots display the predicted mean functional difficulty scores for ADL and IADL over 17 waves (2020-2024). Shaded areas indicate the 95% CI. Trends were estimated using linear mixed-effects models adjusted for baseline age, gender, education, housing type, marital status, number of chronic diseases, social support, isolation, and engagement, as well as cognitive scores. The β coefficient with 95% CI represents the change in functional difficulty over time for either ADL or IADL. ADLs = Activities of Daily Living, IADLs = Instrumental Activities of Daily Living.

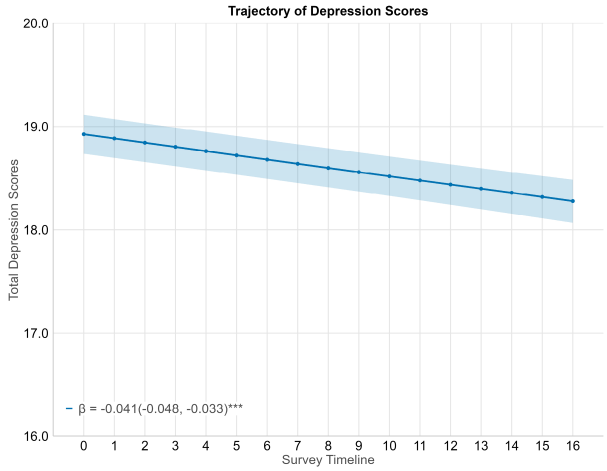

Figure S4. Overall trajectories of depression scores over 17 observation points (2020-2024). Shaded areas indicate the 95% CI. Trends were estimated using linear mixed-effects models adjusted for baseline age, gender, education, housing type, marital status, number of chronic diseases, social support, isolation, and engagement, as well as cognitive scores. The β coefficient with 95% CI represents the change in depression scores over time.
